# FEDERAL NUTRITION POLICIES AND PROGRAMS

**DOI:** 10.1093/geroni/igz038.260

**Published:** 2019-11-08

**Authors:** Robert Blancato

**Affiliations:** Matz, Blancato & Associates, Washington, District of Columbia, United States

## Abstract

This paper will discuss the Older Americans Act nutrition programs and their reauthorization progress, older adult programs under the US Department of Agriculture, and advances in nutrition services being made in healthcare programs such as Medicare Advantage and managed long-term care services and supports. It will also discuss funding for federal older adult nutrition programs and their sustainability moving forward.

